# A comparison of biologically variable ventilation to recruitment manoeuvres in a porcine model of acute lung injury

**DOI:** 10.1186/1465-9921-5-22

**Published:** 2004-11-24

**Authors:** Duane J Funk, M Ruth Graham, Linda G Girling, James A Thliveris, Bruce M McManus, Elizabeth KY Walker, Edward S Rector, Craig Hillier, J Elliott Scott, W Alan C Mutch

**Affiliations:** 1 Department of Anaesthesia, University of Manitoba, Winnipeg, Manitoba, Canada; 2 Department Human Anatomy and Cell Science, University of Manitoba, Winnipeg, Manitoba, Canada; 3 James Hogg iCapture Centre, McDonald Research Laboratories, University of British Columbia, Vancouver, British Columbia, Cananda; 4 Department of Immunology, University of Manitoba, Winnipeg, Manitoba, Canada; 5 Department of Oral Biology, University of Manitoba, Winnipeg, Manitoba, Canada

## Abstract

**Background:**

Biologically variable ventilation (return of physiological variability in rate and tidal volume using a computer-controller) was compared to control mode ventilation with and without a recruitment manoeuvre – 40 cm H_2_O for 40 sec performed hourly; in a porcine oleic acid acute lung injury model.

**Methods:**

We compared gas exchange, respiratory mechanics, and measured bronchoalveolar fluid for inflammatory cytokines, cell counts and surfactant function. Lung injury was scored by light microscopy. Pigs received mechanical ventilation (F_I_O_2 _= 0.3; PEEP 5 cm H_2_O) in control mode until PaO_2 _decreased to 60 mm Hg with oleic acid infusion (PaO_2_/F_I_O_2 _<200 mm Hg). Additional PEEP to 10 cm H_2_O was added after injury. Animals were randomized to one of the 3 modes of ventilation and followed for 5 hr after injury.

**Results:**

PaO_2 _and respiratory system compliance was significantly greater with biologically variable ventilation compared to the other 2 groups. Mean and mean peak airway pressures were also lower. There were no differences in cell counts in bronchoalveolar fluid by flow cytometry, or interleukin-8 and -10 levels between groups. Lung injury scoring revealed no difference between groups in the regions examined. No differences in surfactant function were seen between groups by capillary surfactometry.

**Conclusions:**

In this porcine model of acute lung injury, various indices to measure injury or inflammation did not differ between the 3 approaches to ventilation. However, when using a low tidal volume strategy with moderate levels of PEEP, sustained improvements in arterial oxygen tension and respiratory system compliance were only seen with BVV when compared to CMV or CMV with a recruitment manoeuvre.

## Background

A negative consequence of mechanical ventilation using lower tidal volumes (V_T_) in patients with acute lung injury (ALI) or acute respiratory distress syndrome (ARDS) is alveolar collapse [1-3]. Numerous strategies to recruit these collapsed units have been advocated, but the efficacy of various recruitment manoeuvres for improving and sustaining gas exchange is controversial. Increased PEEP levels have been advocated to maintain patency of the recruited lung, but higher levels of PEEP can cause regional overinflation [4], potentially contributing to ventilator associated lung injury [5]. Moreover, recent evidence finds that when patients with ALI/ARDS are managed with a low tidal volume (V_T_) approach the addition of higher PEEP levels offers no further improvement in outcome [6]. Thus, high levels of PEEP may no longer have the same relevance for ALI/ARDS management as before. Independent of increases in F_I_O_2_, it remains unclear how best to improve and sustain oxygenation, during low V_T _ventilation strategies for ALI/ARDS management.

Buchman [7] and others [8,9] have highlighted how decreased physiological variability can negatively impact critically ill patients. When such patients require assisted ventilation, physiological variability or "noise" can be restored to the respiratory rate and V_T _through use of biologically variable ventilation (BVV), a unique computer-controlled version of control mode ventilation (CMV). With BVV, gas exchange and respiratory mechanics improved in animal models, with [10] and without PEEP [11], during low V_T _protocols using an ARDSNet algorithm [12] and in healthy lungs during prolonged ventilation under anaesthesia [13]. After deliberate collapse with one lung ventilation, recruitment was accelerated [14]. A recent clinical trial showed BVV improved gas exchange and respiratory mechanics in patients undergoing abdominal aortic aneurysmectomy [15]. Other investigators showed noisy ventilation increased surfactant phospholipid levels compared to CMV [16] and a mathematical model of how BVV can enhance recruitment and gas exchange has been advanced [17]. While previous work has indicated BVV results in superior gas exchange and respiratory mechanics compared to CMV with added sighs, this was a post-hoc comparison in a model of deliberate alveolar collapse [14], not a model of ALI/ARDS. As well, the sigh breaths were not equivalent to the larger sustained breaths customarily seen with a recruitment manoeuvre. Thus, it remains unknown if BVV is inferior, comparable or superior to conventional low V_T _ventilation with a recruitment manoeuvre in ALI/ARDS using a low V_T _approach.

Therefore, in this study in pigs with oleic acid lung injury, we compared BVV to conventional CMV or CMV with a recruitment manoeuvre (CMV-RM) of 40 cm H_2_O of continuous positive airway pressure for 40 sec performed hourly for 5 hrs. This approach has been shown to improve oxygenation in patients with early ARDS who do not have any chest wall impairment [18]. A multimodal approach was used to compare the three ventilation strategies. We measured gas exchange and respiratory mechanics. Bronchoalveolar lavage (BAL) fluid was collected to determine cell counts, inflammatory mediators and surfactant function. Tissue was examined by light microscopy to assess lung injury with an established scoring system [19,20] at end experiment.

## Methods

### Experimental Preparation

Pigs (weighing 20–30 kg) received 0.6 mg atropine, 15 mg midazolam, and 300 mg ketamine intramuscularly for sedation. Isoflurane 5% in 100% oxygen was delivered via facemask to induce anaesthesia. When sufficient depth of anaesthesia was achieved, the pigs were intubated orotracheally with a 6.0 mm cuffed endotracheal tube. Mechanical ventilation was instituted with an Ohio 7000 ventilator (Ohio Medical, Madison WI) with minute ventilation adjusted to maintain a PaCO_2 _of 35–45 mm Hg. Anaesthesia was maintained with 2% isoflurane in 100% oxygen during surgical preparation. Intravenous rocuronium bromide (1 mg/kg/hr) was administered by continuous infusion for muscle relaxation. Lactated Ringer's solution was given intravenously during the surgical preparation and for the duration of the experiment.

A thermodilution pulmonary artery catheter (7.5-Fr) was inserted and advanced into the right external jugular vein until a satisfactory pulmonary capillary wedge tracing was obtained. Temperature was measured from the tip of the pulmonary artery catheter. The right femoral artery was cannulated for continuous pressure transduction and arterial blood gas (ABG) analysis. A 5-Fr single lumen femoral venous catheter was advanced into the inferior vena cava for infusion of oleic acid. A surgical tracheotomy was performed and the animal was switched to an Esprit^® ^ventilator (Respironics Inc., Palo Alto CA) capable of delivering either CMV or BVV. The ventilator was set to deliver a square wave inspiratory flow pattern with an I:E ratio of 1:2. Isoflurane was discontinued, and a propofol/ketamine infusion at 10/2.5 mg/kg/hr substituted to maintain anaesthesia.

After a 30 cm H_2_O recruitment manoeuvre for 30 sec, animals were ventilated with a V_T _of 10 ml/kg at an F_I_O_2 _of 0.3, with 5 cm H_2_O of PEEP. Respiratory rate was adjusted to maintain PaCO_2 _between 35–45 mm Hg.

After 15 min to stabilize, baseline measurements were obtained. Haemodynamic measurements included mean arterial pressure (MAP), heart rate, central venous pressure (CVP), mean pulmonary artery pressure (MPAP), and pulmonary artery occlusion pressure (PAOP). All haemodynamic data were continuously recorded on a Gould 2600 Oscillograph (Gould, Cleveland, OH). Cardiac output (CO) was measured by thermodilution, in triplicate, at stated measurement periods.

A pneumotachograph (Model 3700; Hans Rudolph, Kansas City, MO) with the sensor immediately proximal to the tracheotomy was used to measure airway pressures and V_T _intermittently; this data was recorded using an advanced CODAS (Dataq Instruments, Akron, OH) data acquisition system.

Arterial and mixed venous gases were analyzed using a Radiometer ABL 500 (Copenhagen NV, Denmark). Arterial and mixed venous oxygen content, oxygen saturation, and haemoglobin concentrations were measured with a Radiometer OSM3 set for porcine blood.

Static respiratory system compliance (Crs) was measured in triplicate by clamping the expiratory limb of the ventilator circuit at the end of inspiration for 1 sec to obtain a plateau pressure. The V_T _used was that which the animal was receiving at that time.

### Oleic Acid Lung Injury

Oleic acid was infused via the 5-Fr femoral venous catheter at 0.2 ml/kg/hr until PaO_2 _<60 mm Hg for two consecutive measurements (PaO_2_/F_I_O_2 _<200). Dopamine was started at 5 μg/kg/min and was titrated to keep the MAP >60 mmHg. When the oxygenation target was achieved, the oleic acid infusion was stopped and the PEEP was increased to 10 cm H_2_O. Ten minutes after the increase in PEEP, an arterial blood gas sample was obtained to determine if PaO_2 _had increased. PaO_2 _had to be >75 mm Hg but <90 mm Hg. This was considered to represent adequate lung injury, but indicate that collapsed alveoli could be recruited with the additional PEEP. If the PaO_2 _did not increase, the experiment was terminated. If the PaO_2 _increased to >90 mm Hg, additional oleic acid was infused until the PaO_2 _decreased to <60 mm Hg.

### Ventilation Protocol

A low V_T _(7 ml/kg) protocol was initiated and the respiratory rate increased to 30 bpm. After 10–15 min arterial blood gas sampling was done to assess the stability of the PaO_2_. If the PaO_2 _remained stable, the animals were then randomized into one of three groups: conventional ventilation with a V_T _of 7 ml/kg (CMV); conventional ventilation with V_T _of 7 ml/kg with a 40 sec, 40 cm H_2_O recruitment manoeuvre performed hourly (CMV-RM). The recruitment manoeuvre was performed at end-expiration with the PEEP level maintained at 10 cm H_2_O at an F_I_O_2 _of 0.3; or biologically variable ventilation (BVV) with a mean V_T _of 7 ml/kg.

Following stable oleic acid lung injury, haemodynamic, gas exchange and respiratory system compliance (Crs) measurements were recorded and obtained hourly thereafter. Measurements in the CMV-RM group were obtained 5 min after each recruitment manoeuvre (RM). An additional measure of gas exchange and Crs was made in the CMV-RM group 50 min after the RM, to ascertain the duration of effect of the RM.

### Wet:Dry Lung Weight Ratios

At the end of the experiment, a sternotomy was performed. Animals were sacrificed with a lethal dose of thiopental. The trachea was then clamped at end inspiration and the heart and lungs were removed *en bloc*. Following removal, the lungs were suspended upside down for 10 min to collect bronchoalveolar (BAL) fluid. Samples of BAL fluid were collected in heparinized saline and then frozen immediately at -80^o^C. Fluid was then sent for cytokine analysis, measures of surfactant function and flow cytometry. The lungs and previously collected BAL fluid were weighed and the lungs were suspended and aerated overnight. The following day, the lungs were placed in an oven to dry, and following a stable dry measurement, wet:dry weight ratio was calculated.

### Surfactant Function Assays

Surfactant function was assessed on BAL fluid samples using a capillary surfactometer (Calmia Medical, Toronto, Canada) in the manner of Enhorning and colleagues [21,22]. Such an approach can delineate differences in surfactant function with lung inflammation [23]. Surfactometry was performed under two conditions:

1. Raw BAL fluid surfactant function analysis – centrifuging at 200 g for 5 min to rid large debris, then the supernatant spun at 10,000 g and pellet resuspended in 100 μL saline and analyzed using the surfactometer.

2. Surfactant resuspended in 100 μL of saline after chloroform/methanol extraction. Chloroform/methanol extraction permits the lipid and phospholipid fraction to dissolve in the organic nonpolar solvent (chloroform) and the solvent evaporated to dryness. Volume of the final ratio for chloroform:methanol:water was 1:1:0.9. Following extraction, the pellet was resuspended and analyzed using the surfactometer.

The percentage of time that the capillary tube was open for 2 min was determined for each sample, in triplicate, then averaged. Standards were saline; 0% patent for a 2 min time period and bovine surfactant; >98% patent for a 2 min time period. Each sample was measured in a blinded fashion in both conditions.

### Flow Cytometry Analysis

The BAL fluid sample was passed through a cell strainer and 100 μL was transferred into a 5 × 75 mm tissue culture tube. Red cells were lyses by the addition of 500 μL Optilyse C (Beckman Coulter, Mississauga, Canada) and following 15 min incubation at room temperature, the cell suspension was diluted by the addition of 500 μL Isoton II and 100 μL FlowCount Fluorospheres (Beckman Coulter). The cell suspension was immediately analyzed on a Beckman Coulter EPICS Altra cell sorter configured with a high-speed quartz flow cell tip and a water-cooled argon laser emitting 150 mW at 488 nm. Forward and side light scatter signals were employed to derive 2-parameter histograms which clearly defined the fluorescent beads and three populations of cells, subsequently defined as mononuclear cells, neutrophils and eosinophils. At least 20,000 cells were analyzed for each sample. The acquisition software provided with the instrument automatically calculated the concentrations of each cell population based on the number of events in each of the 4 analysis regions.

### Cytokine Assays for IL-8 and -10 ELISA

The incubation times and washes were preformed as specified in each respective kit: (BioSource International, Camarillo, CA). Immunoassay kits for swine IL-8 and IL-10 were used. Sample incubation times were kept as constant as possible by pre-plating on a blank 96 well plate before transferring to the coated assay plate. ELISA plates were read at 450 nm by an SLT Rainbow plate reader (Lab Instruments, Research Triangle Park, NC). Standard curves and concentration calculations were performed according to kit directions.

### Histological Assessment

For light microscopy, four blocks of tissue from the right lung – upper lobe, middle lobe, nondependent and dependent lower lobe, fixed in buffered formalin, processed and embedded in formalin. Sections were cut and stained with haematoxylin and eosin. Under light microscopy, a lung injury scoring system modified from Rotta et al. [19,20] was used, based on the following variables: alveolar and interstitial inflammation, alveolar and interstitial haemorrhage, oedema, atelectasis and necrosis. The severity of injury was graded for each of the seven variables: no injury = 0; injury to 25% of the field = 1; injury to 50% of the field = 2; injury to 75% of the field = 3; and diffuse injury = 4; for a maximal total score of 28.

### Statistical Analysis

Parametric data were analyzed by repeated measures ANOVA using least squares means test matrices to identify differences within and between groups from group × time or group effects. Bonferroni's correction was applied where appropriate. Non-parametric comparison of the lung injury scores was by Kruskal-Wallis test. In all circumstances a p-value ≤0.05 corrected for multiple comparisons was considered significant.

## Results

Twenty-five experiments were undertaken. One animal was discarded because oleic acid injury could not be established after 1.5 hrs of infusion. In the CMV-RM group, one animal died 2 hr into the experiment but data are included in analysis in this experiment up to time of the animal's demise. Complete experiments were done in twenty-three animals; (n = 8 for all three groups).

### Temperature and Haemodynamic Data

Temperature and haemodynamic data are shown in Table 1: see Additional File 1. All data reported as mean ± SD, unless stated. A significant group × time interaction was seen for temperature. A crossover in temperature was seen with greater temperature at baseline in the BVV group that decreased over time by a mean of 0.8°C by end experiment. In contrast in both of the CMV groups the temperature increased modestly. Least squares means test showed MAP lower in CMV-RM at one hr and beyond after oleic acid compared to the other 2 groups. MPAP was increased significantly following administration of oleic acid in all groups. Between groups MPAP was lower with BVV from 1 hr after oleic acid. No group × time interaction was seen for PAOP (p = 0.755) but by least squares means test at baseline, PAOP was lower in the BVV group at baseline and at one hr following oleic acid. In all groups CO decreased following oleic acid and remained depressed beyond (group × time interaction p = 0.521).

### Respiratory Gas and Derived Data

**Figure 1:**
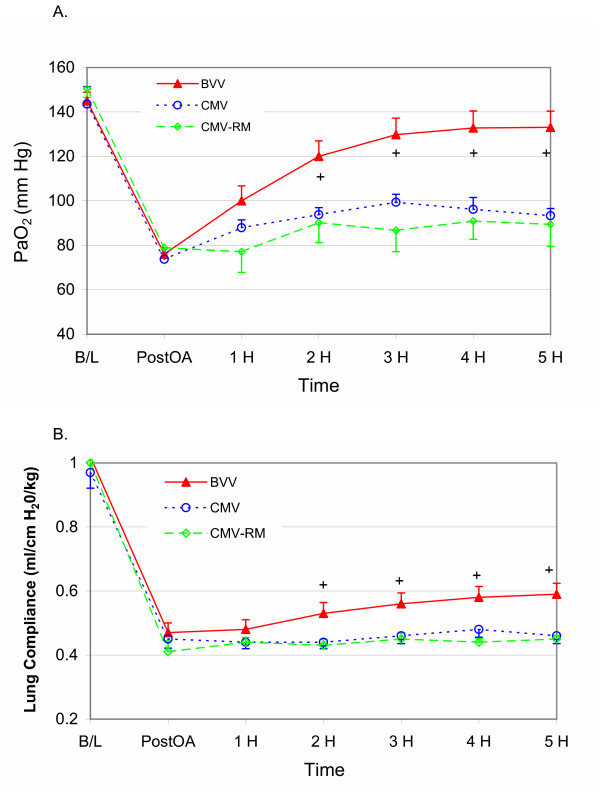
**1a and b – Arterial Oxygenation and Respiratory System Compliance. **Arterial oxygen tension (PaO_2_) over time for the 3 groups (BVV in red; CMV in blue and CMV-RM in green) **(a)**. The group × time interaction is p = 0.0001. No difference is seen between groups at baseline and following oleic acid infusion. By 2 hr the PaO_2 _is greater with BVV (+). There is no difference between CMV and CMV-RM. Respiratory system compliance over time for the 3 groups **(b)**. The group × time interaction is p = 0.089. Least squares means tests revealed BVV had significantly greater compliance after 2 hr (+). There was no difference between CMV and CMV-RM.

Significant differences were seen between groups for PaO_2 _over time (Figure 1a). The group × time interaction was p = 0.0001 with greater PaO_2 _seen with BVV, compared to either CMV or CMV-RM. There was no difference in PaO_2 _between groups at baseline, following oleic acid administration or during the first hour of the experiment. A clear separation in PaO_2 _levels became apparent after 2 hr following oleic acid injury in the BVV group. There was no difference between CMV and CMV-RM over time. Respiratory system compliance for the 3 groups is shown in Figure 1b. The group × time interaction was p = 0.089 between groups. Comparison of compliance between groups with least squares means showed significant differences between BVV with CMV and CMV-RM after 2 hr.

Table 2: see Additional File 2, shows respiratory gas and derived data for each group. There was no group × time interaction for PaCO_2 _(p = 0.660). In all groups, the PaCO_2 _increased following oleic acid injury and was essentially stable at these elevated levels for the remainder of the experiment. The group × time for PvO_2 _was significant at p = 0.002 with significantly lower PvO_2 _over time in the CMV-RM group. Shunt fraction was lower in the BVV group (group × time interaction p = 0.003).

### Recruitment Effects on Oxygenation and Compliance

PaO_2 _tended to decrease immediately following a recruitment manoeuvre, but increased over time as measured at 50 min. PaO_2 _significantly decreased in RM4 and 5 (-11.1 ± 5.2 mm Hg and -5.2 ± 4.4 mm Hg respectively). Following recruitment, PaO_2 _increased slowly early in the experiment at RM2 and RM3 (10.1 ± 5.5 mm Hg and 11.8 ± 9.1 mm Hg respectively), but failed to do so in the later time periods. For respiratory system compliance, no statistically significant differences were seen at any time period over the course of the experiment in the CMV-RM group.

### Airway Pressure Data

A group × time interaction for mean Paw was seen (p = 0.002) with Paw lowest with BVV (Paw at 5 hr was 14.3 ± 0.4 cm H_2_O with BVV; 15.1 ± 0.2 with CMV-RM and 15.1 ± 1.0 with CMV respectively). In all 3 groups, mean Paw increased following oleic acid. A more pronounced effect was seen with peak Paw (group × time; p = 0.0001) with mean peak pressures over time least with BVV (peak Paw at 5 hr was 23.2 ± 1.9 cm H_2_O with BVV; 28.8 ± 0.8 with CMV-RM and 28.6 ± 2.8 with CMV respectively). There was no group × time interaction for V_T _in ml/kg measured over time (p = 0.617).

### Lung Histology

When the lung was scored in the upper, middle, nondependent and dependent lower lobes in the right lung, no differences were seen for any region by Kruskal-Wallis test between the 3 groups. In the dependent region of the lower lobes, injury was considerable with scores ranging between 9/28 and 22/28. Wet:dry weight ratios for the three groups were BVV; 8.5 ± 4.0, CMV; 8.9 ± 1.2, and CMV-RM; 11.5 ± 4.8; not significant between any group.

### Capillary Surfactometry

**Figure 2:**
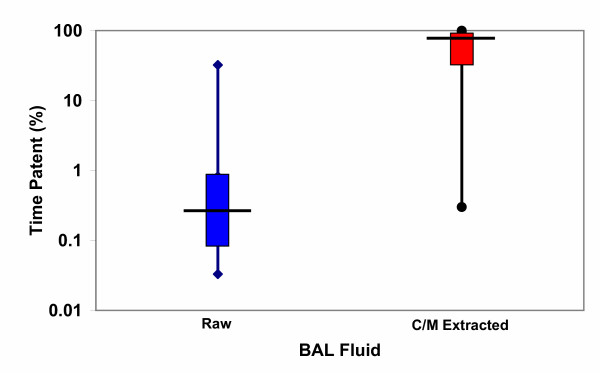
**Capillary Surfactometry. **Box and whisker plots for capillary surfactometry results from both raw (blue) and chloroform/methanol extracted (red) BAL fluid and patency time (%) over 2 min – log scale. In most circumstances the raw BAL fluid has minimal surface activity – low capillary patency. The one animal with a high raw value was from the BVV group. This patency markedly improved with extraction suggesting reactivation of surfactant with removal of BAL oedema fluid, proteins, including cytokines and cellular debris. In 4 animals there was essentially no change in function with extraction. There were 2 animals in the CMV group, and one each in the BVV and CMV-RM in this population. No statistically different behaviour in surfactant function was seen between groups, either for raw or extracted surfactant function.

Capillary surfactometry was done on raw BAL fluid and following chloroform/methanol extraction. There was no difference in surfactometry for raw BAL fluid between groups; the duration of capillary tube patency as an index of surfactant function was: 6.7 ± 14.3%, 0.7 ± 0.8% and 0.9 ± 1.7%; for BVV, CMV and CMV-RM respectively – not significant. Following chloroform/methanol extraction, surfactant function of the same fluid usually improved dramatically – see Figure 2. Here, duration of capillary patency was 57.6 ± 40.1%, 47.4 ± 43.8% and 81.3 ± 33.8%; for BVV, CMV and CMV-RM respectively – not significant between any groups. In 4 animals, the chloroform/methanol extraction had no discernible effect on surfactant function, in all others surfactant function improved markedly.

### Flow Cytometry

The flow cytometry of BAL fluid indicate no significant difference between cell counts either in terms of neutrophils, monocytes or eosinophils between groups. Neutrophils were the predominant cell type, at 74% ± 15% of the totals. The total BAL cell counts were highly variable however, with neutrophil counts varying from a low of 187 to a high of 26,189 cells/μL – a 140-fold difference. There was no group effect for neutrophils; p = 0.741. The mean BAL neutrophil counts were 8237 ± 8738/μL for BVV, 12024 ± 12328/μL for CMV and 6492 ± 4219/μL for CMV-RM. BAL fluid volume was also measured based on gravity drainage from the lung over a 10 min period. Volume was not significantly different between groups and was 13.6 ± 16.8 mL with BVV, 15.1 ± 10.6 mL with CMV and 28.4 ± 23.2 mL with CMV-RM. The total neutrophil count in the BAL fluid was calculated as neutrophils/μL × BAL volume in μL for each animal. Mean total neutrophil counts were 1.38 × 10^8 ^for BVV, 1.82 × 10^8 ^for CMV and 2.34 × 10^8 ^for CMV-RM. There was no significant difference between groups.

### ELISA Results and Cytometry Correlations

The ELISA results to assess the cytokine IL-8 concentration in BAL fluid indicated no statistically significant difference for the 3 groups: 564 ± 551 pg/mL with BVV; 653 ± 639 pg/mL with CMV and 300 ± 235 pg/mL with CMV-RM. As in the measurement of neutrophil counts, there was significant variability with IL-8 levels ranging over a 385-fold concentration difference. Such a broad range of data in both neutrophil counts and IL-8 concentration suggested the possibility of power law behaviour and correlation between these variables. A very strong power law relationship was found between IL-8 pg/mL and absolute neutrophil count/mL for pooled data; y = 77609x^0.75^; R^2 ^= 0.85, n = 13. This power law relationship exceeded the linear (R^2 ^= 0.60) and exponential (R^2 ^= 0.41) curve fits.

The number of mononuclear cells from cell cytometry of BAL fluid was not different between groups: for BVV, 1346 ± 743 cells/μL; for CMV, 1767 ± 905 cells/μL; and for CMV-RM, 1068 ± 579 cells/μL. These cells presumably in large part represent alveolar macrophages. A power law relationship was seen between pooled data for a correlation between IL-8 concentration in pg/mL and monocyte count/mL – 1.0 × 10^-6^x^1.38^; n = 13, R^2 ^= 0.83. In this situation the linear and exponential fits were R^2 ^= 0.61 and R^2 ^= 0.69 respectively. In this analysis, IL-8 concentration is presumed to be the dependent variable – being released by the monocytes (counts on the x-axis).

The IL-10 results from BAL fluid, as well, indicate no statistically significant difference for the 3 groups: 119 ± 173 pg/mL for BVV; 124 ± 146 pg/mL for CMV; and 149 ± 168 pg/mL for CMV-RM. Unlike IL-8 concentrations the range of variation for IL-10 concentrations was significantly less – the maximum range differing by only 16-fold. There was no power-law relationship found between IL-10 and absolute monocyte count for pooled data: y = 7.5x^0.15^; n = 13, R^2 ^= 0.07.

## Discussion

The main finding in this study of acute lung injury in a porcine model is that BVV significantly improved oxygenation and respiratory mechanics with no difference in indices of lung injury, inflammation or surfactant function compared to the more conventional ventilation techniques – CMV or CMV with a standard recruitment manoeuvre. The improvements seen with BVV were sustained over the course of the experiment; an effect not seen with recruitment. Such improvement was obtained at similar measured mean V_T _but at lower peak and mean airway pressure and greater respiratory system compliance.

These findings are similar to previously published results from our laboratory showing that BVV was superior to both CMV and CMV with sigh breaths. These sigh breaths were of no advantage in a model of healthy lung recruitment – lung reinflation after a controlled collapse for one hr with contralateral one-lung ventilation [14]. In that model, the sigh breaths were not as large or as sustained as the recruitment manoeuvre studied here. An important difference from our previous work is that the current experiment examined recruitment in a low V_T _model of ALI/ARDS; not in healthy lung. As well, we have confirmed the differences between BVV and CMV seen in other work from the laboratory using the same model [12]. No important differences were seen for gas exchange or respiratory mechanics between CMV and CMV-RM in the current study.

Shunt fraction was significantly lower with BVV. Lynch et al. [24] and Sandoval et al. [25] showed that shunt fraction is directly related to cardiac output and PvO_2_. Thus, lower PvO_2 _in the CMV-RM group should have minimized shunt but here the shunt fraction is greatest. The lowest shunt with BVV indicates that the numerator of the shunt equation is less in this group, indicating enhanced blood flow in aerated lung units.

A multimodal approach to assess lung injury, lung inflammation and surfactant function indicated no discernable differences between the 3 approaches to ventilation:

1. Light microscopy studies demonstrated no significant differences between groups for lung injury in any region examined – from upper to dependent lower lobes. This finding indicates no difference between groups in lung injury as assessed by histology, with the caveat that only small areas of lung in each region were examined in a condition known to be heterogeneous.

2. No difference in surfactant function was seen between groups as assessed by capillary surfactometry. Following oleic acid lung injury, surfactant function was markedly depressed when compared to a surfactant standard – see Figure 2. The raw surfactant from the BAL fluid was not usually active beyond a few percent of normal function, with no difference between groups. In contrast, following chloroform/methanol extraction, surfactant function improved toward normal in most circumstances, again with no difference between groups. It is well known that surfactant is inactivated in the presence of oedema fluid, inflammatory proteins and cellular debris, but that the surfactant can be reactivated in the right circumstances as seen here following chloroform/methanol extraction. No correlation was seen for surfactant function of pooled data for PaO_2_, wet:dry weight ratios, respiratory system compliance, or peak airway pressure. Arold et al. [16] have shown that noisy ventilation similar to BVV can result in greater surfactant phospholipid concentration over time in healthy lungs. We do not address this specific issue.

3. No difference between groups is seen for cell counts in the BAL fluid. There is considerable variation in this analysis and a full data set is not present. A significant number of studies of cell count could not be undertaken because of the nature of the BAL fluid. When thick with proteinaceous debris the samples often were unable to be prepared for meaningful cytometry analysis. Of the data represented, no difference in the proportion of neutrophils, monocytes or eosinophils is apparent for any of the 3 approaches to ventilation. Allen and Bates [26] have shown that the number of neutrophils in BAL fluid – a 500 fold difference between groups in their study – did not correlate with changes in respiratory mechanics as assessed by changes in elastance with deep inflation in mice. Their findings suggest that the non significant differences in neutrophils counts – 70% greater total counts with CMV-RM than BVV – should be of no influence on respiratory mechanics.

4. IL-8 is considered to be the major neutrophil chemoattractant cytokine in lung diseases like ARDS [27]. IL-10 markedly inhibits lymphocyte and phagocytic function, essential for an adequate immune response to invading microbes [28]. Initial IL-10 serum levels have been shown to be significantly higher in patients with ARDS who died as compared to survivors [29]. No differences were seen between groups in the levels of the inflammatory cytokines IL-8 and IL-10 in BAL fluid. This is not surprising given similar monocyte and neutrophil counts between groups. A large coefficient of variation for both cytokine concentrations is demonstrated, a common finding in most such studies [30]. Such large variation makes meaningful comparisons between groups problematic. A power law relationship between IL-8 concentrations and monocyte and neutrophil counts in BAL fluid is a new insight. We found a very positive correlation relating IL-8 concentration and cell counts. The markedly variable IL-8 concentrations and cell counts in BAL fluid under controlled experimental conditions, suggests a highly nonlinear process has been initiated following oleic acid administration. Despite the observed variation in cytokine and cell counts a strong linkage is suggested by the power-law correlation. This observation, in and of itself is not surprising, given that IL-8 is a known chemoattractant. But the power law descriptor suggests that both low and high concentrations of IL-8 and corresponding cell counts are more frequent than expected. These data also suggest that this interaction is "scalable" with no specific mean value to be anticipated [31]. In this circumstance, large coefficients of variation are to be expected, making meaningful standard comparisons between groups problematic. Looking for similar power law correlations may help to elucidate the nature of the inflammatory process in the future. We have seen differences in temperature between BVV and CMV in the past with greater IL-8 levels with CMV [12]. We cannot confirm that finding in this study. As well, we were unable to confirm a previous observation of an inverse correlation of IL-8 concentration and wet:dry weight ratios in this model (R^2 ^= 0.007 in this experiment, n = 24). Failure to reconfirm these findings may, in part relate to the nonlinear relationships highlighted above. Small changes in initial conditions may preclude similar findings at end experiment.

The above observations, collectively, indicate no fundamental differences between the ventilation strategies studied in regards oleic acid or superimposed ventilator associated lung injury. No one technique seems clearly advantageous. However, BVV alone improves oxygenation significantly following acute lung injury over time, an effect that was sustained, suggesting it is the best technique of the 3 studied to recruit atelectatic lung as assessed by greater PaO_2 _and respiratory system compliance. BVV has been shown to recruit in a pure model of lung collapse – re-expansion of lung following cessation of one lung ventilation [14]. With BVV, the addition of a noisy end-inspiratory pressure has been advanced as the mechanism to improve oxygenation in the face of the nonlinear characteristics of alveolar recruitment [17].

It could be argued that we have not chosen the optimal recruitment strategy to compare to the two other modes of mechanical ventilation. That said, we have chosen a well recognized approach to recruitment [18], one associated with improved patient outcome in a carefully conducted clinical trial. Amato and colleagues [32] did not state how often their chosen recruitment manoeuvre was used in their clinical study – just that it was used frequently – usually in association with suctioning the tracheobronchial tree. We have applied the Amato recruitment strategy in a comprehensive manner – hourly in an acute model of ARDS. But, in contrast to Amato and colleagues, where PEEP was increased from 5 cm H_2_O to 2 cm H_2_O above P_flex _in an attempt to prevent derecruitment, we maintained PEEP at 10 cm H_2_O throughout. In a porcine model of lung lavage, lung remained recruited if PEEP was at 10 cm H_2_O: the value chosen in this study [33]. This level of PEEP is that recommended in a recent review of mechanical ventilation in ARDS, especially in the context of patchy ARDS where the risk of overdistention of patent alveoli increases the potential for volutrauma [34]. Furthermore, in the ARDSNet study, the Amato recruitment manoeuvre failed to sustain an improved arterial oxygenation, so was abandoned [6]. Failure to demonstrate an increase in oxygen with recruitment suggests either redistribution of blood flow to poorly ventilated regions or potentially increased alveolar flooding with alveolar-capillary disruption. Such causes may account for our failure to demonstrate improved oxygenation at 5 min after individual recruitment manoeuvres. Positron emission tomography indicates that PaO_2 _will not increase if the sustained inflation does not restore aeration to the atelectatic regions because a significant fraction of pulmonary blood flow is shunted to nonaerated regions [35]. Such may be the case in this study as shunt fraction was greatest in the CMV-RM group. Fujino and colleagues [36] have shown that a recruitment manoeuvre similar to the Amato manoeuvre – 40 cm H_2_O for 60 sec – was not beneficial in another animal model and that a more aggressive recruitment manoeuvre – PEEP 40 cm H_2_O and pressure control ventilation of 20 cm H_2_O, with a respiratory rate of 10, and I:E ratio 1:1 for 2 min – was successful only after the second hour. Allen et al. [37] have shown in a mouse model with deep inflation that PEEP at various, albeit low levels (1, 3, 6 cm H_2_O) did not influence the time constants for recovery in elastance both before and after lung lavage. They conclude that for a deep inflation to be beneficial in their model that it may be necessary to apply the inflation several times a minute. Thus, the appropriate PEEP level, with and following recruitment, remains controversial [38,39], but what is clear from the current study is that at the same PEEP level significantly better gas exchange and respiratory mechanics occurs with BVV over either mode of CMV – with or without recruitment, with no differences in lung injury, inflammation or surfactant function.

**Figure 3:**
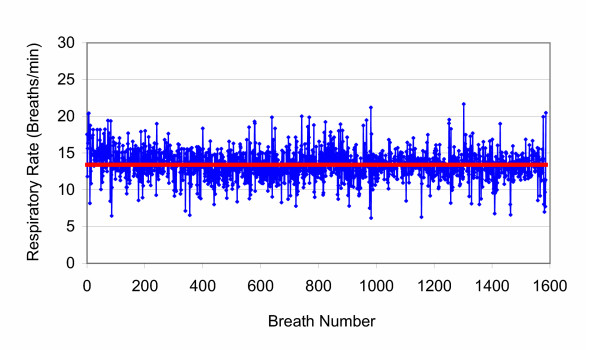
**Human Respiratory Rate Variability File. **Human breathing variability file used for the study. This is the raw data from a spontaneously breathing healthy female volunteer. The mean rate was 13.4 ± 2.0 breaths/min (shown as the red line). There are 1587 breaths in this file. With BVV, the ventilator is configured as a volume divider at a fixed minute ventilation so that respiratory rate × tidal volume product is constant. Thus the breath-by-breath volume related to instantaneous respiratory rate obtained from sequentially reading the above file in any given experiment is obtained from the minute ventilation/[(instantaneous breath rate/13.4) × chosen mean rate]. Analysis reveals that these data have fractal characteristics.

The human variability file for respiratory rate used to program the ventilator is shown in Figure 3. Mathematical analysis indicates that this file is not fundamentally different in characteristics from previous files used to program the ventilator. Such variability files have fractal time sequences and are associated with health. Whether or not "fractal noise" is superior to random or "white noise" is as yet unproven, however, such fractal variability is characteristic of human health. Suki et al. [17] showed the importance of noise to increase the volume recruited when alveoli are collapsed and provide a mathematical framework for how BVV can result in better gas exchange and respiratory mechanics in the context of alveolar collapse. This study confirms previous findings that BVV can deliver the same minute ventilation over time at lower mean peak airway pressure and greater respiratory compliance.

## Conclusions

This study shows that BVV with a human variability file was superior to either CMV or CMV with a recruitment manoeuvre (CMV-RM) for sustained improvement in gas exchange and respiratory mechanics in a porcine model of acute lung injury. Prior work has demonstrated an advantage of BVV in a clinical setting of atelectasis [15]. The current study suggests that BVV may be superior to more conventional approaches to improve oxygenation without increasing the risk of volutrauma when low V_T _strategies are used for management of ALI/ARDS. A well accepted recruitment strategy has been compared to BVV in this animal study. It is possible that other approaches for recruitment could be superior to the one chosen. However, all chosen modes of recruitment would increase airway pressures over and above those seen with BVV, potentially increasing the risk of volutrauma.

## Authors' contributions

D.J.F. was responsible for conduct of the experiments as a fellow in the Anesthesia Laboratory. M.R.G. supervised conduct of the experiments, helped analyze data and helped write the paper. L.G.G. helped with the experiments, data retrieval and collation and table and figure production. J.A.T. did the histological assessments and analysis. B.M.M. supervised the cytokine assays and their interpretation and helped write the paper. E. K-Y. W. conducted the cytokine assays. C.H. did the surfactant assays while a fellow in the Oral Biology Laboratory. J.E.S. supervised and interpreted the results of the surfactant assays. W.A.C.M. conceived the study, analyzed and interpreted data and helped write the paper.

## Supplementary Material

Additional File 1 Table 1: Temperature and Haemodynamics Click here for file

Additional File 2 Table 2: Respiratory Gas and Derived Data Click here for file
