# Supplementary material for: A comparison of biologically variable ventilation to recruitment manoeuvres in a porcine model of acute lung injury
Source: Respir Res. 2004 Nov 24;5(1):22. doi: 10.1186/1465-9921-5-22 (PMC535805; doi:10.1186/1465-9921-5-22)
Supplement: Additional File 1 — Table 1: Temperature and Haemodynamics [file 1465-9921-5-22-S1.pdf]

Table 1

Temperature and Haemodynamics

	Pre OA	Post OA	1 h	2 h	3 h	4 h	5 h
Temp							
BVV	37.0 ± 0.5 +	37.1 ± 0.7	37.1 ± 0.8	36.6 ± 1.0 *	36.5 ± 1.1 *	36.3 ± 1.3 *	36.2 ± 1.3 *+
CMV-RM	36.3 ± 1.0	36.6 ± 1.2	37.0 ± 1.2	36.6 ± 1.1	36.5 ± 1.0	36.6 ± 1.3	36.8 ± 0.8
CMV	36.8 ± 0.8	37.0 ± 1.1	37.0 ± 0.9 *	37.0 ± 1.1	37.0 ± 1.2	37.1 ± 1.2	37.1 ± 1.0
MAP							
BVV	86 ± 15	85 ± 11	97 ± 11	105 ± 16 *	101 ± 14	97 ± 21	101 ± 22
CMV-RM	90 ± 16	75 ± 8	77 ± 17+	84 ± 20 +	84 ± 19 +	77 ± 26 +	83 ± 33 +
CMV	86 ± 15	87 ± 18	100 ± 21 *	112 ± 20 *	104 ± 17	103 ± 28	104 ± 16 *
MPAP							
BVV	17.8 ± 3.3	32.0 ± 3.7 *	30.0 ± 3.6 *+	27.8 ± 4.3 *+	27.1 ± 3.9 *+	27.4 ± 4.0 *+	27.0 ± 4.4 *+
CMV-RM	18.4 ± 1.8	34.4 ± 4.4 *	34.8 ± 3.3 *	32.9 ± 3.6 *	32.2 ± 4.6 *	31.3 ± 4.4 *	32.0 ± 7.7 *
CMV	20.1 ± 2.7	37.3 ± 8.5 *	36.4 ± 6.4 *	36 ± 5.4 *	34.3 ± 7.6 *	34.3 ± 5.5 *	36.9 ± 8.4 *
PAOP							
BVV	8.3 ± 1.7 +	10.3 ± 1.7 *	9.8 ± 2.3 +	10.1 ± 2.1 *	10.3 ± 1.6 *	9.9 ± 2.2	9.6 ± 2.3
CMV-RM	9.8 ± 1.6	10.9 ± 1.5	12.3 ± 3.6 *	11.6 ± 1.1	11.1 ± 1.4	10.8 ± 1.2	10.3 ± 1.5
CMV	9.5 ± 1.6	11.5 ± 1.4 *	11.9 ± 2.7 *	11.0 ± 1.1	11.0 ± 1.1	10.6 ± 0.9	11.5 ± 1.4 *
CO							
BVV	4.5 ± 0.5	3.3 ± 0.7 *	3.1 ± 0.7 *	2.9 ± 0.7 *	3.0 ± 0.5 *	3.0 ± 0.6 *	3.0 ± 0.5 *
CMV-RM	4.1 ± 0.6	2.6 ± 0.8 *	2.2 ± 0.7 *+	2.3 ± .07 *	2.2 ± 0.9 *+	2.5 ± 0.9 *	2.5 ± 1.0 *
CMV	4.4 ± 1.5	3.1 ± 1.0 *	2.9 ± 0.9 *	2.7 ± 1.1 *	2.9 ± 1.3 *	3.1 ± 1.7 *	3.4 ± 1.9 *

MAP = Mean Arterial Pressure (mm Hg)
MPAP = Mean Pulmonary Arterial Pressure (mm Hg)
PAOP = Pulmonary Artery Occlusion Pressure (mm Hg)
CO = Cardiac Output (L/min)

* P< .05 Within Groups
+ P< .05 Between Groups
