# Supplementary material for: A comparison of biologically variable ventilation to recruitment manoeuvres in a porcine model of acute lung injury
Source: Respir Res. 2004 Nov 24;5(1):22. doi: 10.1186/1465-9921-5-22 (PMC535805; doi:10.1186/1465-9921-5-22)
Supplement: Additional File 2 — Table 2: Respiratory Gas and Derived Data [file 1465-9921-5-22-S2.pdf]

Table 2

Respiratory Gas and Derived Data

	Pre OA	Post OA	1 h	2 h	3 h	4 h	5 h
$PaCO_2$							
BVV	39.7 ± 3.5	53.4 ± 7.1 *	59.8 ± 11.0 *	54.3 ± 9.4 *	52.7 ± 7.2 *	52.2 ± 6.3 *	52.0 ± 6.8 *
CMV-RM	38.3 ± 3.8	49.9 ± 3.6 *	54.1 ± 6.1 *	55.2 ± 11.0 *	51.5 ± 6.8 *	49.9 ± 7.0 *	49.4 ± 6.2 *
CMV	40.0 ± 2.8	53.9 ± 7.1 *	55.8 ± 9.5 *	54.2 ± 9.4 *	50.8 ± 7.9 *	49.7 ± 7.9 *	48.9 ± 6.8 *
PvO_2							
BVV	42.2 ± 3.7	39.0 ± 2.7 +	40.7 ± 4.2	41.3 ± 5.2	40.5 ± 4.4	39.9 ± 3.5	39.5 ± 2.4
CMV-RM	43.8 ± 5.8	37.0 ± 4.7 *	34.8 ± 8.8 *+	36.2 ± 6.8 *	35.6 ± 4.7 *	35.0 ± 7.7 *+	32.3 ± 8.4 *+
CMV	42.2 ± 4.0	38.3 ± 3.9 *	39.1 ± 3.5	38.3 ± 4.2 *	37.9 ± 4.2 *	38.1 ± 2.4 *	38.1 ± 3.2 *
pHa							
BVV	7.45 ± .03	7.32 ± .05 *	7.29 ± .07 *	7.32 ± .07 *	7.34 ± .06 *	7.35 ± .06 *	7.35 ± .07 *
CMV-RM	7.46 ± .03	7.33 ± .04 *	7.26 ± .08 *	7.31 ± .05 *	7.33 ± .04 *	7.35 ± .04 *	7.34 ± .03 *
CMV	7.46 ± .03	7.33 ± .04 *	7.26 ± .08 *	7.35 ± .03 *	7.33 ± .04 *	7.35 ± .04 *	7.34 ± .03 *
Q_S/Q_T							
BVV	3.0 ± 1.9	22.1 1 6.1 *	15.1 ± 8.7 *	7.8 ± 4.3	6.7 ± 4.7 +	5.4 ± 3.7	6.5 ± 5.0 +
CMV-RM	3.6 ± 3.9	22.0 ± 8.3 *	24.3 ± 17.6 *	16.4 ± 8.5 *	16.8 ± 9.8 *	13.2 ± 6.8 *	15.4 ± 9.2 *
CMV	4.1 ± 2.4	23.7 ± 5.8 *	14.2 ± 4.9 *	12.9 ± 6.6 *	12.8 ± 6.5 *	11.1 ± 4.9 *	12.8 ± 5.4 *

$PaCO_2$ = Arterial Partial Pressure CO_2 (mm Hg)
PvO_2 = Mixed Venous Partial Pressure O_2 (mm Hg)
pHa = Arterial pH
Q_S/Q_T = Shunt Fraction (%)

* $p < .05$ Within Groups
+ $p < .05$ Between Groups
